# Observational study on wearable biosensors and machine learning-based remote monitoring of COVID-19 patients

**DOI:** 10.1038/s41598-021-82771-7

**Published:** 2021-02-23

**Authors:** Ka-Chun Un, Chun-Ka Wong, Yuk-Ming Lau, Jeffrey Chun-Yin Lee, Frankie Chor-Cheung Tam, Wing-Hon Lai, Yee-Man Lau, Hao Chen, Sandi Wibowo, Xiaozhu Zhang, Minghao Yan, Esther Wu, Soon-Chee Chan, Sze-Ming Lee, Augustine Chow, Raymond Cheuk-Fung Tong, Maulik D. Majmudar, Kuldeep Singh Rajput, Ivan Fan-Ngai Hung, Chung-Wah Siu

**Affiliations:** 1 Cardiology Division, Department of Medicine, The University of Hong Kong, Hong Kong SAR, China; 2 Biofourmis Singapore Pte. Ltd, Singapore, Singapore; 3 Harmony Medical Inc, Hong Kong SAR, China; 4 Infectious Diseases Division, Department of Medicine, The University of Hong Kong, Hong Kong SAR, China

**Keywords:** Viral infection, Predictive medicine

## Abstract

Patients infected with SARS-CoV-2 may deteriorate rapidly and therefore continuous monitoring is necessary. We conducted an observational study involving patients with mild COVID-19 to explore the potentials of wearable biosensors and machine learning-based analysis of physiology parameters to detect clinical deterioration. Thirty-four patients (median age: 32 years; male: 52.9%) with mild COVID-19 from Queen Mary Hospital were recruited. The mean National Early Warning Score 2 (NEWS2) were 0.59 ± 0.7. 1231 manual measurement of physiology parameters were performed during hospital stay (median 15 days). Physiology parameters obtained from wearable biosensors correlated well with manual measurement including pulse rate (*r* = 0.96, *p* < 0.0001) and oxygen saturation (*r* = 0.87, *p* < 0.0001). A machine learning-derived index reflecting overall health status, Biovitals Index (BI), was generated by autonomous analysis of physiology parameters, symptoms, and other medical data. Daily BI was linearly associated with respiratory tract viral load (*p* < 0.0001) and NEWS2 (*r* = 0.75, *p* < 0.001). BI was superior to NEWS2 in predicting clinical worsening events (sensitivity 94.1% and specificity 88.9%) and prolonged hospitalization (sensitivity 66.7% and specificity 72.7%). Wearable biosensors coupled with machine learning-derived health index allowed automated detection of clinical deterioration.

## Introduction

Coronavirus Disease 2019 (COVID-19) due to severe acute respiratory syndrome coronavirus-2 (SARS-CoV-2) has spread at an alarming rate and resulted in a pandemic causing more than 12 million infections and 560,000 deaths globally as of July 12 2020^1–4^. The overwhelmingly large number of COVID-19 patients, together with the shortage of hospital beds and isolation facilities beleaguered healthcare systems worldwide^5–9^. Despite having initially mild symptoms, COVID-19 patients may still deteriorate during hospital stay; for instance, one-third of COVID-19 patients with mild or moderate disease on admission deteriorated and required mechanical ventilation in intensive care units^1,3,10,11^. Thus, early recognition of patient deterioration is crucial for successful clinical management. Conventionally, hospitalized patients are assessed intermittently by manual measurement of physiology parameters and early warning score systems such as National Early Warning System 2 (NEWS2) are used to identify deteriorating patients (Appendix Table 2)^12–14^. Amidst the pandemic, manual measurement of physiology parameters by healthcare workers inevitably increases their exposure to the virus and risk of contracting COVID-19, especially with the constrained personal protective equipment (PPE) supply^15,16^. In fact, as of April 8 2020, 22,073 healthcare workers from 52 countries were infected by COVID-19 according to situation report issued by the World Health Organization (WHO)^17^.

Biosensors for physiology parameters have been progressively miniaturized in recent years to allow incorporation into wearable devices such as armbands and wristbands, enabling monitoring of heart rate, heart rate variability, respiration rate, oxygen saturation, blood pulse wave, skin temperature and actigraphy in a continuous and autonomous manner^18–23^. As state-of-the-art telecommunication technologies allow instantaneous and multi-directional massive data transfer at low cost, it is now possible to remotely monitor a large number of patients in a real-time^24^, and to relay information to clinicians for timely intervention. Conceivably, in the setting of COVID-19, remote patient monitoring tools enable healthcare workers to manage infected patients with minimal physical contact, thereby minimizing their risk of getting infected, as well as reducing the consumption of PPE. Furthermore, machine learning-based analytic systems may enhance clinical management by detecting early signs of clinical deterioration from continuous streams of physiology data collected from wearable biosensors, allowing prompt and precise delivery of intervention, and optimization of resources use. In this study, we explored the potential of utilizing wearable biosensors and machine learning-based remote monitoring platform for managing COVID-19 patients hospitalized in isolation wards.

## Methods

### Study design and participants

This observational study was conducted in the Department of Medicine, Queen Mary Hospital, The University of Hong Kong, Hong Kong. The study aimed to evaluate the feasibility of utilizing wearable biosensors and machine learning-based analysis of multivariate longitudinal physiology parameters in the management of COVID-19 patients. Patients hospitalized with PCR confirmed COVID-19 were recruited. Patients were excluded if they were under 18 years of age, had NEWS2 > 4 on admission, required intensive care on admission, or lacked skills in operating simple electronic devices. In addition to the standard management, study participants were continuously monitored using wearable biosensors and machine learning-based remote monitoring platform during hospital stay. Written informed consent were obtained from all recruited participants. The study protocol was approved by the institutional review board of the University of Hong Kong/Hospital Authority Hong Kong West Cluster and registered in the clinicaltirals.gov (NCT04343794). This clinical trial protocol follows the Standard Protocol Items: Recommendations for Interventional Trials (SPIRIT)^25,26^. The underlying protocol follows the Consolidated Standards of Reporting Trials (CONSORT)^27,28^.

### Isolation ward setting and routine monitoring

All COVID-19 patients were initially managed in isolation wards and were transferred to intensive care unit or stepdown wards when necessary. Depending on the initial clinical condition, COVID-19 patients in isolation wards had their physiology parameters monitored every 4-hourly to 12-hourly by healthcare workers wearing recommended PPE.

### Wearable biosensors-based remote monitoring platform

**Figure 1:**
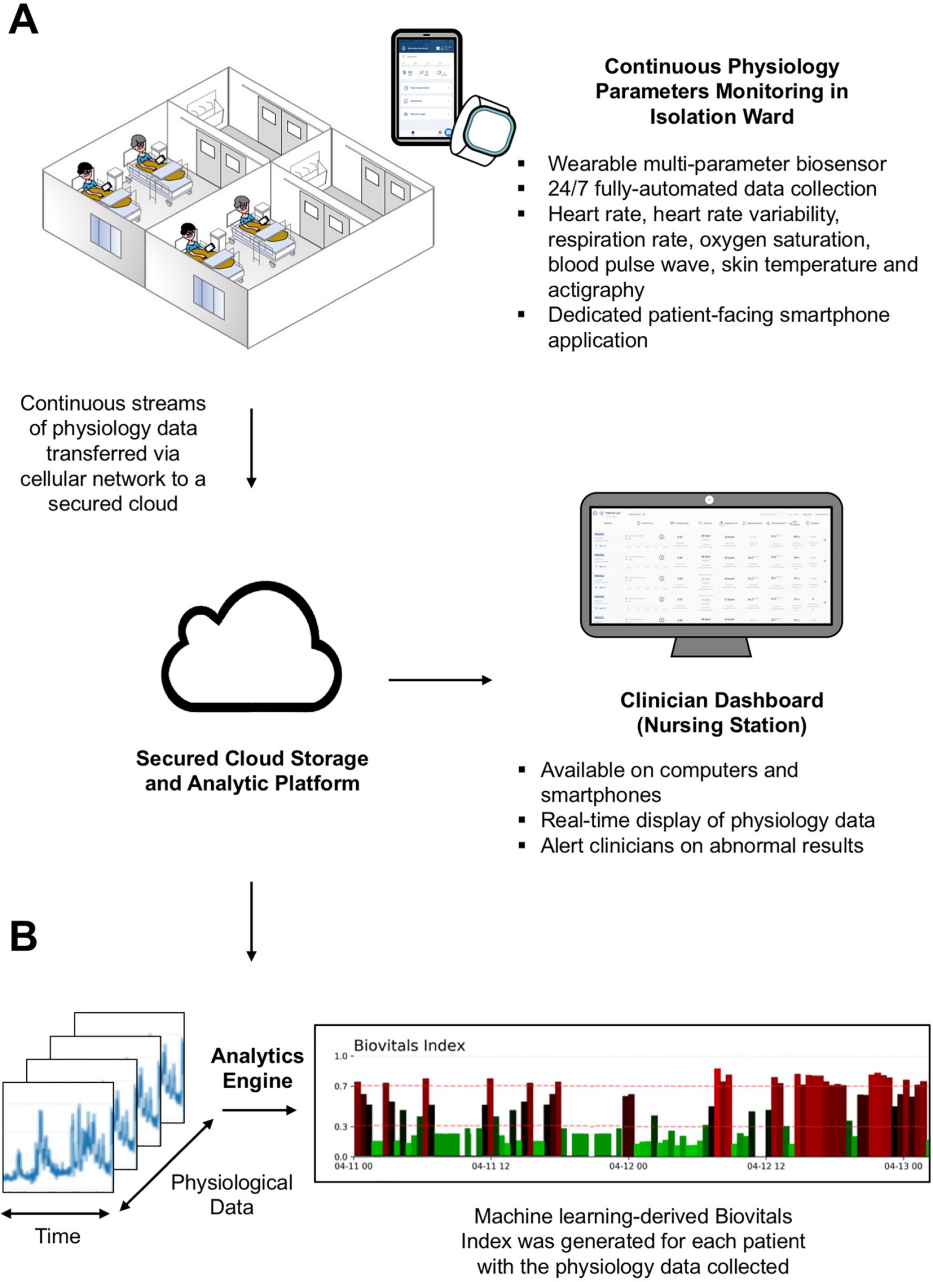
(**A**) Isolation ward setting and study design. (**B**) Machine learning-based derivation of Biovitals Index with continuous streams of physiology data from wearable biosensors.

Patients were remotely monitored using Biovitals Sentinel Platform (Biofourmis, Boston, MA, USA), which consisted of wearable biosensors, patient-facing smartphone application, secured cloud for data hosting and processing, and web-based dashboard for clinicians. (Fig. 1) The wearable biosensor, Everion (Biofourmis, Boston, MA, USA), was a continuous physiology parameters monitoring device worn on the upper arm to capture multi-dimensional physiology parameters including heart rate, heart rate variability, respiration rate, oxygen saturation, blood pulse wave, skin temperature and actigraphy. Patients were instructed to wear the wearable biosensors for 23 h per day and charge them while showering. The wearable biosensors continuously transmitted physiology data via Bluetooth to paired smartphones. In addition, patients were instructed to report their symptoms using the dedicated smartphone application. All data were automatically transferred in real-time to a secured cloud for hosting and analysis via cellular network. Physiology data, patient-reported symptoms and machine-learning derived analytic results were displayed on a web-based dashboard at the nursing station and the clinicians’ office for healthcare workers to review.

### Machine learning for COVID-19 progression monitoring

The cloud based analytical system used, Biovitals Analytics Engine (Biofourmis, Boston, MA, USA), was a machine learning system developed as a disease-specific predictive management tool which received 510(k) clearance from the U.S. Food and Drug Administration as a medical device for ambulatory physiology monitoring^29^. One of its output, the Biovitals Index, was a machine learning-derived health index that reflected the overall health status of users. It was generated by autonomous analysis of physiology parameters, symptoms, and other medical data^29^ (Fig. 1). Higher Biovitals Index reflected worse health status and a longer duration of further hospital stay is expected, and vice versa. The machine learning and statistical methods for derivation of Biovitals Index were described in previous publication^29^, Supplementary Appendix and Appendix Table 1.

### Measures and outcomes

The primary virologic outcome was the viral load from respiratory sample estimated semi-quantitatively using RT-PCR cycle threshold (Ct) value for SARS-CoV-2, which was sampled every 2–4 days in the isolation wards after admission. The viral load was classified as “high” with Ct value ≤ 20, “medium” with Ct value 20–30, “low” with Ct value > 30, and “undetected”. The clinical status was assessed on admission and every morning during the hospital stay using NEWS2, which is endorsed by the British National Health System for initial assessment and serial monitoring of patients with sepsis to early detect clinical deterioration^30^ (Appendix Table S2). Clinical worsening event was defined as the new occurrence of one or more of the followings: (1) NEWS2 ≥ 5, (2) individual component parameter constituting NEWS2 = 3, (3) increase in daily NEW2 ≥ 3, (4) use of oxygen therapy, and (5) intensive care unit admission. COVID-19 patients were discharged from the hospital after fulfilling the current discharge criteria including (1) clinical criteria, that is when the clinical conditions of the patients improve and afebrile for at least 24 h and (2) laboratory evidence of SARS-CoV-2 clearance as evidenced by negative RT-PCR tests for nasopharyngeal swab with sampling interval ≥ 24 h.

### Statistical analysis

Continuous and discrete variables were expressed as mean ± standard deviation and percentages, respectively. Chi-square test or Fisher’s exact test was used to compare categorical variables between groups. Student's *t* test or Mann–Whitney *U* test was performed to compare continuous variables. Pearson correlation test was used for evaluating the linear association between two variables. Analysis of variance (ANOVA test) was used to compare the sample mean between multiple groups. Receiver operating characteristics (ROC) curve was used for evaluating the trade-off between sensitivity and specificity. Area under the curve (AUC) of ROC was used for evaluating the predictive accuracy for classification models. All tests were two-sided, and a *p*-value < 0.05 was considered significant. All statistical analyses were performed using SPSS software (version 21.0), R (version 3.6.1), and Python (version 3.6) programming languages.

## Results

Between March 19, 2020 and April 11, 2020, 37 patients with PCR-confirmed COVID-19 were admitted to the isolation wards of Queen Mary Hospital. 34 patients fulfilling the inclusion and exclusion criteria were recruited. The median age was 32 years (IQR 21–50 years), and 52.9% were men. Table 1 summarized the clinical characteristics of the study population. On admission, 19 (55.9%) patients presented with fever, and 13 (38.2%) had cough. The median systolic and diastolic blood pressure were 138 mmHg (121–153 mmHg) and 85 mmHg (77–92 mmHg) respectively, and the median heart rate was 90 bpm (81–99 bmp). No study participant required oxygen supplement on admission, and the median SpO_2_ was 98% (97–99%). The mean NEWS2 score was 0.59 ± 0.7. All patients were managed in negatively pressured and double-doored cubicles within isolation wards. Six patients received a combination of interferon beta-1b, lopinavir-ritonavir and ribavirin; 9 patients received lopinavir-ritonavir and ribavirin; 13 patients received hydroxychloroquine and interferon beta-1b; and 6 patients received supportive care only. No patients received corticosteroid in the study.

**Table 1:** Clinical characteristics of the study population.

Clinical characteristics	(n = 34)
**Demographics and co-morbidities**	
Age, years	32 (21–50)
Male, n (%)	18 (52.9)
Hypertension, n (%)	2 (5.9)
Diabetes mellitus, n (%)	3 (8.8)
Stroke, n (%)	1 (2.9)
**Symptoms**	
Fever, n (%)	19 (55.9)
Cough, n (%)	13 (38.2)
Sore throat, n (%)	7 (22.2)
Sputum, n (%)	6 (17.6)
Shortness of breath, n (%)	2 (5.9)
Vomiting, n (%)	1 (2.9)
Diarrhea, n (%)	11 (11.3)
Fatigue, n (%)	3 (8.8)
Myalgia, n (%)	3 (8.8)
Loss of taste and/or smell, n (%)	8 (23.5)
**Vital measurement**	
Systolic blood pressure, mmHg	138 (121–153)
Diastolic blood pressure, mmHg	85 (77–92)
Heart rate, bpm	90 (81–99)
SpO_2_, %	98 (97–99)
NEWS2 score	0.59 ± 0.7
**Chest radiographic findings**	
Normal	8 (23.5)
Unilateral infiltrates	15 (44.1)
Bilateral infiltrates	11 (32.4)
**Laboratory findings**	
White cell counts, 1 × 10^9^/ml	6.4 ± 2.2
Lymphocyte counts, 1 × 10^9^/ml	1.6 ± 0.6
Hemoglobin, g/dl	14.3 ± 1.8
Platelet counts, 1 × 10^12^/ml	257 ± 69
Alanine aminotransferase U/l	30.8 ± 23.1
Creatinine, μmol/L	77.8 ± 14.5
Elevated troponin level, n (%)	1 (2.9)

### Physiology parameters monitoring

During the median hospital stay of 15 days (9–20 days), a total of 1231 manual measurement of physiology parameters were made for these 34 patients, which consumed 747 sets of PPE. As expected, the time-stamped physiology parameters including heart rate and oxygen saturation captured using wearable biosensors correlated with those from manual measurement with Pearson correlation coefficients (r) of 0.96 (95% CI 0.95–0.97), and 0.87 (95% CI 0.81–0.91) respectively (Appendix Figure 1).

### Correlation between Biovitals Index, NEWS2 and viral load

**Figure 2:**
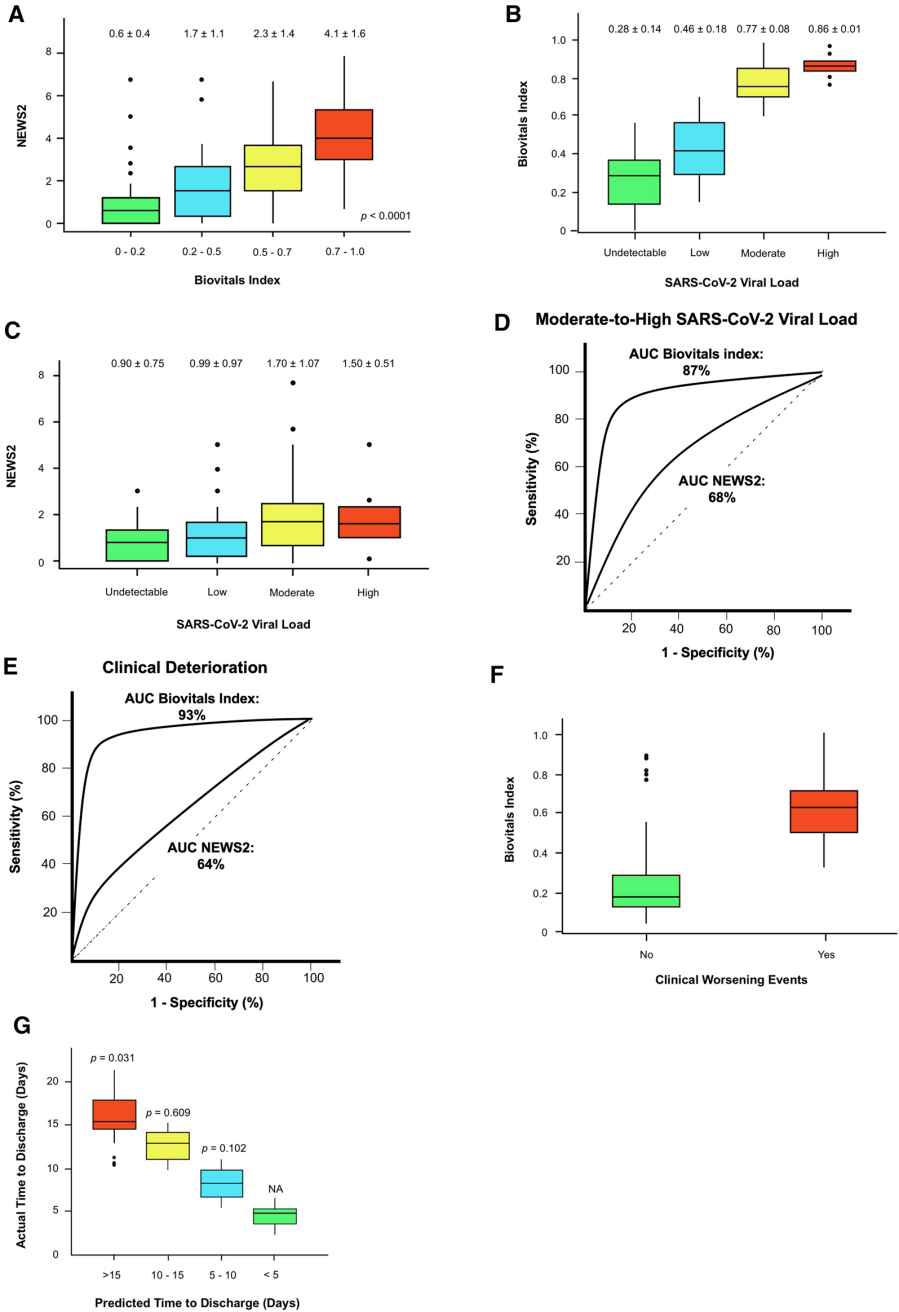
(**A**) 24-h average Biovitals Index positively correlated with 24-h NEWS2. (**B**,**C**) Respiratory samples SARS-CoV-2 viral load determined by RT-PCR cycle threshold (Ct) values correlated with 24-h average Biovitals Index (*p* < 0.0001) but did not correlate with 24-h NEWS2 (*p* = 0.004, r = 0.15). (**D**) The area under the curve of 24-h average Biovital Index to identify moderate/high viral load was 0.87 (95% CI 0.83–0.90). (**E**,**F**) Biovitals Index predicted clinical worsening events with the area under the curve of 0.93 (95% CI 0.89–0.95) with the optimal cut-off at sensitivity and specificity of 94.1% and 88.9% respectively. 24-h NEWS2 predicted clinical worsening events with area under the curve of 0.64 (95% CI 0.61–0.67) with the optimal cut-off at sensitivity and specificity of 29.4% and 85.7% respectively. (**G**) Actual time-to-discharge in relation to the predicted time-to-discharge derived from machine learning-based projection.

The 24-h average Biovitals Index of individual patients was positively associated with the 24-h NEWS2. (Fig. 2A) The 24-h NEWS2 increased progressively with increasing 24-h average Biovitals Index from 0.6 ± 0.4 with the Biovitals Index between 0.0 and 0.2, to 4.1 ± 1.6 with the Biovitals Index between 0.7 and 1.0 (*p* < 0.0001). To our surprise, the increase in viral load from respiratory samples as determined with RT-PCR Ct value for SARS-CoV-2 was associated with increasing 24-h average Biovitals Index (*p* < 0.0001), but not with the 24-h NEWS2 (*p* = 0.004, r = 0.15). (Fig. 2B,C). Furthermore, the diagnostic performance to identify moderate/high viral load in the respiratory samples was compared between the 24-h average Biovital Index and the 24-h NEWS2. The area under the curve of 24-h average Biovital Index to identify moderate/high viral load was 0.87 (95% CI 0.83–0.90), significantly larger than that of the 24-h NEWS2 (0.68, 95% CI 0.65–0.71). Specifically, the 24-h average Biovitals Index > 0.5 correctly identified 100% moderate/high viral load with a false positive rate of 0.0% and a false negative rate of 11.9%. On the other hand, the 24-h NEWS2 ≥ 5 identified 80% moderate/high viral load with a false positive rate of 0.4% and a false negative rate of 88.6% (Fig. 2D).

### Prediction of clinical worsening events

A total of 17 clinical worsening events occurred in these 34 COVID-19 patients during hospitalization. Biovitals Index alerts detected 16 out of 17 events (94.1%) prior to the clinical events getting noticed in ward with prediction time interval of 21.0 h, ranging from 6 to 39 h. On the other hand, there were 2 Biovitals Index alerts without actual clinical worsening event within the subsequent 3 days (false positive). The area under the curve was 0.93 (95% CI: 0.89–0.95) with the optimal cut-off at sensitivity and specificity of 94.1% and 88.9% respectively. The performance of Biovitals Index to predict clinical worsening events was then compared with the 24-h NEWS2. The area under the curve for 24-h NEWS2 to predict clinical worsening event was only 0.64 (95% CI 0.61–0.67) with the optimal cut-off at sensitivity and specificity of 29.4% and 85.7% respectively (Fig. 2E,F).

### Prediction of length of stay

The Biovitals Index progressively decreased as patients improved. To estimate the remaining length of stay, machine learning-based projection of the Biovitals Index reduction trend was performed. After collecting sufficient data from each patient for fitting into the machine learning model over a variable number of days, one-time predictions of the remaining length of stay were made (Supplementary Appendix and Appendix Figure 2). The median time spent for generating the prediction was 8 days (3–10 days). For COVID-19 patients with an actual total hospital stay ≤ 15 days, there were no statistically significant difference between the actual length of stay and the predicted length of stay. However, for those with actual hospital stay > 15 days, the predicted length of stay was significantly shorter than the actual hospital stay (17.8 days (14–25 days) vs. 21.5 days (16–31 days), *p* = 0.031) (Fig. 2G). Nonetheless, compared with NEWS2 score on admission to predict prolonged hospital stay (> 10 days), the area under the curve of the machine learning model of 0.78 (95% CI 0.72–0.82) with the optimal cut-off at sensitivity and specificity of 66.7% and 72.7%, was superior to the NEWS2 with area under the curve of 0.58 (95% CI 0.53–0.64) with the optimal cut-off at sensitivity and specificity of 51.7% and 59.1%.

## Discussion

In this study we demonstrated feasibility and potential clinical application of an wearable biosensor and machine learning-based remote monitoring platform for managing hospitalized COVID-19 patients. We first showed strong correlation between physiology parameters including heart rate and oxygen saturation obtained from the wearable biosensors and manual measurements. Furthermore, a machine learning-derived health index, Biovitals Index, generated using machine learning-based analysis of multivariate longitudinal physiology parameters demonstrated a superior ability to discriminate COVID-19 patients with moderate/high viral load from those with undetectable or low viral load, to recognize clinical worsening events early, and to predict hospital discharge, than the widely adopted NEWS2.

Prior studies have demonstrated that continuous monitoring is superior to intermittent monitoring in detecting deterioration in hospitalized patients^31–34^. However, continuous monitoring outside the intensive care unit remains uncommon. Setting up continuous monitoring systems used to be cumbersome as it involves connecting patients to sensor devices with numerous electrodes and cables, which restrict patient activities to the bed area. Also, data transmission were highly reliant on in-house telecommunication infrastructure. In contrast, wearable device such as armband or wristband incorporates multiple biosensors in a single form-factor, which allows a higher degree of patient mobility without the constraints of physical wirings. More importantly, data transmission through cellular network avoids the need of installing additional in-house telecommunication infrastructure, allows rapid deployment, and provides versatile and scalable solutions to suit different clinical applications such as emergent response to the COVID-19 pandemic. In this study, the platform covering 4 isolation wards and 2 stepdown wards located in 2 different hospital buildings was successfully deployed within 24 h. Amidst the pandemic, fewer manual measurement of physiology parameters not only lessens the workload of healthcare workers, but also reduces their exposure to the virus, risk of in-hospital infection and consumption of PPE.

One challenge of continuous monitoring of physiology parameters was to determine when to alert clinicians when alarming signs emerge. Conventional remote monitoring systems alert clinicians when any of the monitored physiology parameters exceed pre-determined thresholds and often overload clinicians with false alarms. Early warning systems such as NEWS2 allow more precise deterioration prediction by concurrently considering multiple physiology parameters. In our study, Biovitals Index generated by autonomous analysis of the continuous streams of physiology data predicted clinical deterioration with superior accuracy than the widely adopted early warning system NEWS2, in additional to identifying patients with high viral load. The superior accuracy is partly attributed to the massive amount of physiology parameters available for analysis, which provided a more comprehensive representation of each patient’s health status than intermittent manual measurements. Furthermore, the current system also took historical physiology data of each patient into account and multiple machine learning techniques were used to allow accurate analysis, such data pre-processing, data segmentation, feature extraction, anomaly detection and semi-supervised multivariate regression. Most of the aforementioned elements were not possible with early warning systems like NEWS2. Moving forward, it is possible that machine learning-based analysis of multivariate longitudinal physiology parameters will be increasing utilized in other clinical settings as well. The improved precision in predicting clinical deterioration allows more prompt and targeted intervention to be delivered.

To further validate the value of this wearable biosensor and machine learning-based remote monitoring platform in clinical practice, it is necessary to conduct adequately powered randomized controlled trials to demonstrate its effectiveness in improving patient outcome. In addition to having accurate machine learning-based predictions and alerts, it is also important to appropriately define intervention for each clinical alert. In the setting of COVID-19, as to date since there is still a lack of effective therapeutics, clinical alerts will mostly allow accurate triage of patients and efficient allocation of hospital resources.

### Limitations

This study had several limitations. First**,** it was limited by the relatively small sample size and a single-centred observational design. Second, the study included patients with mild or moderate COVID-19 on admission, limiting the generalizability of the patients with severe or critical disease. Nonetheless, patients with severe or critical COVID-19 are most likely triaged for high dependency units or intensive care units are not the target candidates for remote monitoring. Last but not least, while technically attractive, potential privacy concerns may arise with sensitive physiological data from individual patients being transferred and stored in the networks, necessitating appropriate regulatory measures for proper privacy protect prior to large-scale implementationn^35^.

## Conclusion

Wearable biosensors coupled with machine learning-based multivariate analysis of physiology parameters allowed accurate detection of COVID-19 patients at risk of deterioration.

## Data sharing statement

Deidentified clinical and post-processing physiology data will be available from the corresponding author on reasonable request after obtaining approval by the investigators and signing data access agreement, from date of publication to one year after publication.

## Supplementary Information

Supplementary Information 1.

Supplementary Information 2.

Supplementary Information 3.
