# Supplementary figures and images for: Observational study on wearable biosensors and machine learning-based remote monitoring of COVID-19 patients
Source: Sci Rep. 2021 Feb 23;11:4388. doi: 10.1038/s41598-021-82771-7 (PMC7902655; doi:10.1038/s41598-021-82771-7)

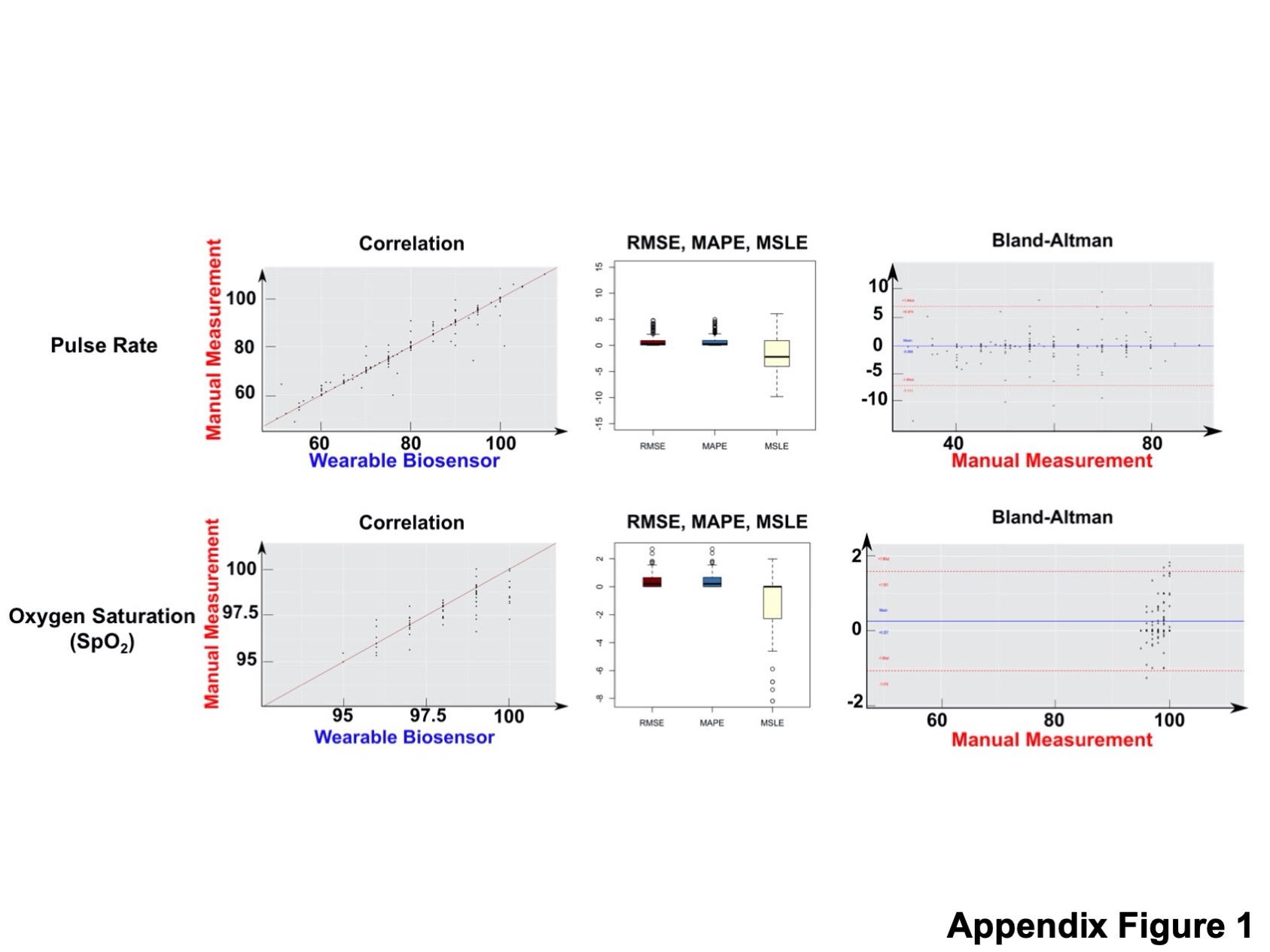

Supplement: Supplementary file 1 — Supplementary Information 1. [file 41598_2021_82771_MOESM1_ESM.jpg]

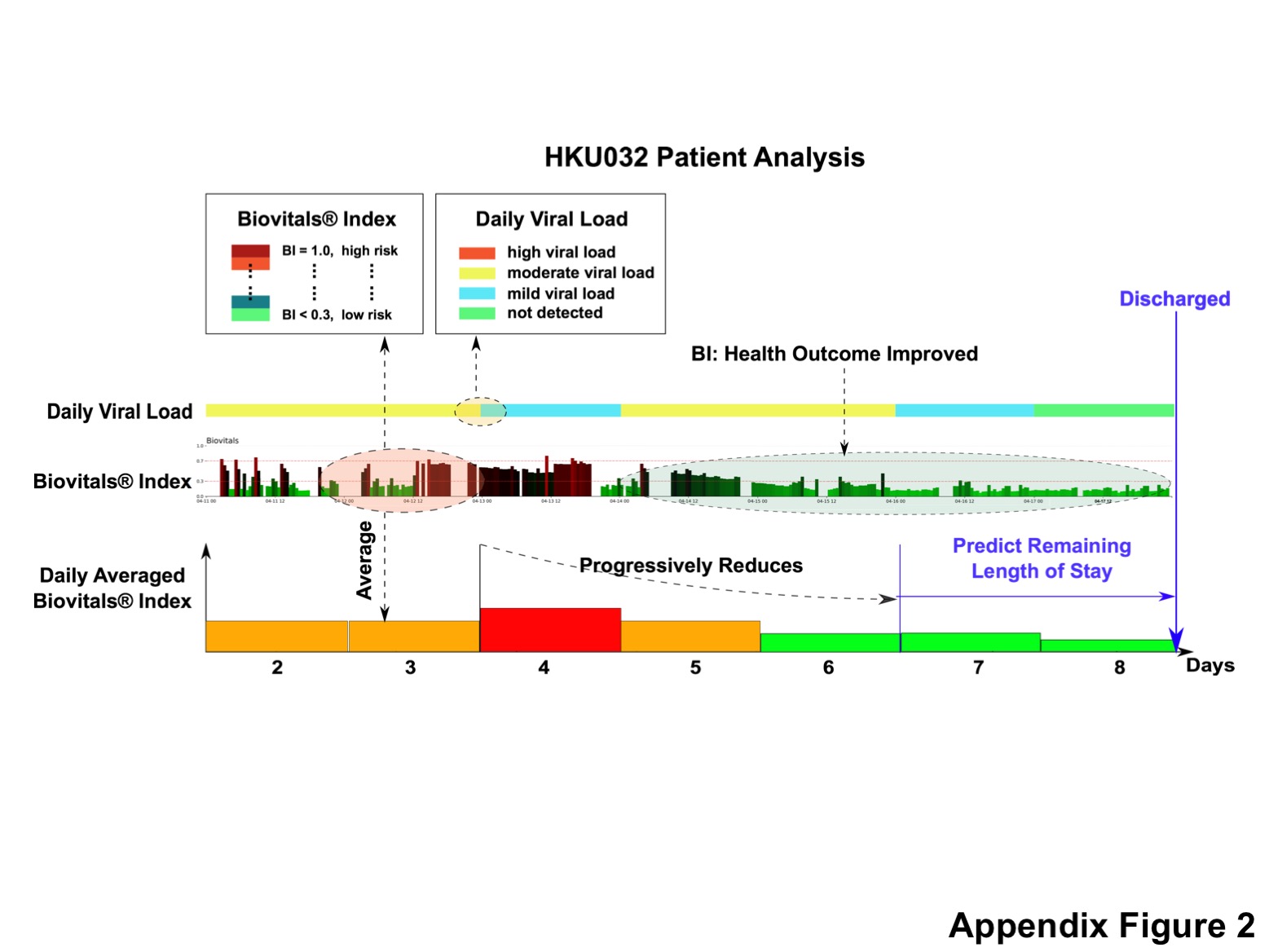

Supplement: Supplementary file 2 — Supplementary Information 2. [file 41598_2021_82771_MOESM2_ESM.jpg]
