# Supplementary material for: Observational study on wearable biosensors and machine learning-based remote monitoring of COVID-19 patients
Source: Sci Rep. 2021 Feb 23;11:4388. doi: 10.1038/s41598-021-82771-7 (PMC7902655; doi:10.1038/s41598-021-82771-7)
Supplement: Supplementary file 3 — Supplementary Information 3. [file 41598_2021_82771_MOESM3_ESM.docx]

**Observational Study on Wearable Biosensors and Machine Learning-based Remote Monitoring of COVID-19 Patients**

^#^Ka-Chun UN, MBBS;^1 #^Chun-Ka WONG, MBBS;^1^ Yuk-Ming LAU, MBBS;^1^ Jeffrey Chun-Yin LEE, MBBS;^1^ Frankie Chor-Cheung TAM, MBBS;^1^ Wing-Hon LAI, PhD;^1^ Yee-Man LAU, PhD;^1^ Hao CHEN, PhD;^2^ Sandi WIBOWO, PhD;^2^ Xiaozhu ZHANG, MS;^2^ Minghao YAN, MS;^2^ Esther WU, PhD;^2^ Soon-Chee CHAN, MS;^2^ Sze-Ming LEE, MBA;^3^ Augustine CHOW, PhD PhD(Bio) DBA EngD;^3^ Raymond Cheuk-Fung TONG, MBBS;^3^ MAJMUDAR Maulik D., MD;^2^ RAJPUT Kuldeep Singh, PhD;^2^ Ivan Fan-Ngai HUNG, MD;^4^ and Chung-Wah SIU, MD.^1^

Institutes: ^1^Cardiology Division, Department of Medicine, The University of Hong Kong, Hong Kong SAR, China; ^2^Biofourmis Singapore Pte. Ltd, Singapore; ^3^Harmony Medical Inc., Hong Kong SAR, China and ^4^Infectious Diseases Division, Department of Medicine, The University of Hong Kong, Hong Kong SAR, China.

^#^These authors contributed equally to this work.

Running Title: Remote Monitoring of COVID-19 Patients
Word count: 2903

**Address of Correspondence:**

Chung-Wah Siu, MD

Cardiology Division, Department of Medicine,

The University of Hong Kong,

Hong Kong SAR, China.

Tel: (852) 2255-4694

Fax: (852) 2818-6304

E-mail: [cwdsiu@hku.hk](mailto:cwdsiu@hku.hk)

**Supplementary Appendix**

**Table of Contents**

Detailed Methods Page 4

Supplementary Tables Page 7

Supplementary Figures Page 10

**Detailed Methods**

**Wearable Biosensor Remote Monitoring Platform**

In addition to the routine clinical monitoring, patients in the isolation wards were continuously monitored using Biovitals Sentinel Platform (Biofourmis, Boston, MA, USA), which consisted of wearable biosensors, patient-facing smartphone application, secured cloud for data hosting and processing, and web-based dashboard for clinicians. The wearable biosensor, Everion (Biofourmis, Boston, MA, USA), was a continuous physiology parameters monitoring device worn on the upper arm to capture multi-dimensional physiology parameters including heart rate, heart rate variability, respiration rate, oxygen saturation, blood pulse wave, skin temperature and actigraphy. Patients were instructed to wear the biosensors for 23 hours per day and charge them while showering. The wearable biosensors continuously transmitted physiology data via Bluetooth to paired smartphones. In addition, patients were instructed to report their symptoms using the dedicated smartphone application. All data were automatically transferred in real-time to a secured cloud for hosting and analysis via cellular network. Physiology data, patient-reported symptoms and machine-learning derived analytic results were displayed on a web-based dashboard at the nursing station and the clinicians’ office for healthcare workers to review.

**Machine Learning for COVID-19 Progression Monitoring**

The cloud based analytical system used, Biovitals Analytics Engine (Biofourmis, Boston, MA, USA), was a machine learning system developed as a disease-specific predictive management tool which received 510(k) clearance from the U.S. Food and Drug Administration as a medical device for ambulatory physiology monitoring.(25) One of its output, the Biovitals Index, was a machine learning-derived health index that reflected the overall health status of users. It was generated by autonomous analysis of physiology parameters, symptoms, and other medical data. Higher Biovitals Index reflects worse health status and a longer duration of further hospital stay is expected, and vice versa.

The machine learning and statistical methods for derivation of Biovitals Index were previously described in detail.(25) Briefly, the Biovitals Index of each patient was initiated by inputting patient demographics, vital signs, selected symptoms, medical history, laboratory and radiology results on admission. Each parameter was assigned a risk score as summarized in Appendix Table 1. A vector consisting of all risk scores was constructed to determine the overall risk level of each patient on admission, which was used to determine the initial value of the Biovitals Index.

The analytic engine then received continuous streams of physiology parameters from the wearable biosensors and symptoms data intermittently reported by patients through the dedicated smartphone application. The massive amount of data was autonomously analysed with machine learning-based data pre-processing, data segmentation, feature extraction, anomaly detection and semi-supervised multivariate regression, to continuously quantify the deviation of the latest health status from baseline, which was then used to update the Biovitals Index.(25)

The Biovitals Index progressively decreased as patients improved. To estimate the remaining length of stay, projection of the Biovitals Index reduction trend was performed by machine learning-assisted time-series data pre-processing, frequency domain analysis, and anomaly detection. After collecting sufficient data from each patient for fitting into the machine learning algorithms over a variable number of days, a one-time prediction of the remaining length of stay was generated.

**Supplementary Tables**

**Appendix Table 1.** Clinical parameters included to derive the baseline Biovitals Index

| **Parameters on admission** | **Data type** | **Risk Score [0 - 1]** |
| --- | --- | --- |
| **Demographics** | | |
| Gender | Categorical | Male: 0.2; Female: 0 |
| Age | Continuous | - |
| **Medical history** | | |
| Atrial fibrillation | Categorical | Yes: 0.5; No: 0 |
| Chronic kidney disease | Categorical | Yes: 0.5; No: 0 |
| Coronary artery disease | Categorical | Yes: 0.7; No: 0 |
| Diabetes mellitus | Categorical | Yes: 0.5; No: 0 |
| Heart failure | Categorical | Yes: 0.7; No: 0 |
| Malignancy | Categorical | Yes: 0.5; No: 0 |
| Smoker | Categorical | Yes: 0.5; No: 0 |
| Number of medical comorbidities | Continuous | - |
| **Vital signs** | | |
| Temperature | Categorical | Febrile: 0.7; Afebrile: 0 |
| Systolic blood pressure | Categorical | Hypertensive or hypotensive: 0.7; Otherwise 0 |
| Diastolic blood pressure | Categorical | Hypertensive or hypotensive: 0.5; Otherwise 0 |
| Oxygen saturation (SpO_2_) | Continuous | - |
| **Symptoms** | | |
| Unconsciousness | Categorical | Yes: 0.7; No: 0 |
| Nausea | Categorical | Yes: 0.5; No: 0 |
| Fatigue | Categorical | Yes: 0.2; No: 0 |
| Dyspnea | Categorical | Yes: 0.7; No: 0 |
| Cough | Categorical | Yes: 0.7; No: 0 |
| **Laboratory and radiology results** | | |
| Hemoglobin | Continuous | - |
| Chest radiograph abnormalities | Categorical | Yes: 0.7; No: 0 |
| Total leukocyte | Continuous | - |
| Lymphocyte | Continuous | - |
| Creatinine | Continuous | - |

**Appendix Table 2.** National Early Warning Score 2 (NEWS2)

|  | 3 | 2 | 1 | 0 | 1 | 2 | 3 |
| --- | --- | --- | --- | --- | --- | --- | --- |
| Pulse (bpm) | ≤ 40 |  | 41-50 | 51-90 | 91-110 | 111-130 | ≥131 |
| Respiratory rate (bpm) | ≤ 8 |  |  |  |  |  | ≥25 |
| Body temperature (°C) | ≤ 35 |  |  |  |  |  |  |
| Systolic BP | ≤ 90 | 91-100 | 101-110 | 111-219 |  |  | ≥220 |
| SpO_2_ (%) scale 1 | ≤ 91 | 92-93 | 94-95 | ≥96 |  |  | ≥97 |
| SpO_2_ (%) scale 2 | ≤ 83 | 84-85 | 86-87 | 88-92 or  ≥93 (Air) | 93-94 (O_2_) | 95-96 (O_2_) | ≥97 (O_2_) |
| Oxygen supplement |  | Yes |  | No |  |  |  |
| Consciousness |  |  |  | Alert | Verbal | Pain | Unresponsive |

The NEWS2 calculation was performed according to the recommendations of The Royal College of Physicians of London.(26) The risk of mortality was stratified as following: aggregate score 1-4 as “low risk”, aggregate score 5-6 or individual parameter = 3 as “medium risk”, and ≥ 7 as “high risk”.(26)

**Supplementary Figures**

**Appendix Figure 1.** Pulse rate and oxygen saturation obtained from wearable biosensors and manual measurement had strong linear association (*p* < 0.0001) with Pearson correlation coefficients (r) of 0.96 (95% CI: 0.95-0.97) and 0.87 (95% CI: 0.81-0.91) respectively. Root mean square error (RMSE), mean absolute percentage error (MAPE), and mean squared logarithmic error (MSLE) of pulse rate and SpO_2_ between wearable biosensors and manual measurement were visualized as boxplots that narrowly centered at 0. The Bland–Altman plots showed agreement between two methods of measurement with the average discrepancy close to 0 and there were no proportional bias.

**Appendix Figure 2.** Biovitals Index progressively decreased as patients improved. To estimate the remaining time of hospital stay required, projection of the Biovitals Index reduction trend was performed by machine learning-assisted techniques.
